# Hand Hygiene Knowledge and Practices Among Visitors to the Holy Masjid (Masjid Al-Haram) During the Month of Ramadan in 2023

**DOI:** 10.7759/cureus.56986

**Published:** 2024-03-26

**Authors:** Amjad S Alzahrani, Turki T Alessa, Heba Y Dosh, Rawan Aljuwaybiri, Wafa A Alshaddadi, Musaad M Almhmadi, Muhammad Irfanullah Siddiqui

**Affiliations:** 1 College of Medicine, Umm Al-Qura University, Makkah, SAU

**Keywords:** pilgrims, knowledge and practices, makkah city, masjid alharam, hand hygiene

## Abstract

Introduction

Visits to the Holy Masjid are considered mass gatherings (MGs), defined as concentrations of people at a specific location for a certain objective over a predetermined amount of time. Such gatherings might strain the host nation’s preparation and reaction capabilities, increasing the chances of spreading infectious diseases.

Aim

To evaluate the comprehension of hand hygiene (HH) and proper HH habits among visitors to the Holy Masjid during the month of Ramadan in 2023.

Methods

A total of 690 visitors to the Holy Masjid were interviewed for this cross-sectional study. The questionnaire was developed using model questions from another published survey.

Results

Of the participants, 541 (78.4%), predominantly female, had generally good knowledge about HH. A total of 282 (40.9%) participants used nothing to clean their hands after shaking hands with someone. Four hundred and eighty (69.6%) participants were aware that poor HH does not spread HIV/AIDS, and 504 (73%) stated that consistent HH does not reduce the body's natural immunity. A total of 530 (76.8%) participants with good knowledge about HH cleaned their hands before meals, compared to 131 (19%) participants with poor knowledge of HH.

Conclusion

Based on the results of our study, the participants' awareness of HH was generally high, with most recognizing the role of good HH in preventing common infectious diseases, such as gastrointestinal and respiratory infections. However, certain aspects of HH, such as the necessity and proper use of alcohol-based hand sanitizers, were not well understood. Regular, focused awareness-raising initiatives are recommended to enhance HH knowledge and practices among visitors to the Holy Masjid.

## Introduction

A mass gathering (MG) is defined by the WHO as a concentration of individuals in a specific place for a specific purpose over a predetermined amount of time, which can burden the host nation's or community’s response and planning capabilities [1]. Each year, millions of Muslims from all over the world come to the Holy Masjid during the Hajj and Umrah in Makkah, with roughly two million pilgrims from 185 countries. The Hajj and Umrah represent two of the largest MGs in the world, performed annually in Mecca, Saudi Arabia. Pilgrims visit various sacred sites around the city of Mecca, and the majority also travel to Medina, the second-holiest site in Islam, to visit the Prophet's Mosque, which contains the tomb of Prophet Muhammad [2-5]. According to information from the Ministry of Hajj and Umrah for internal and external pilgrims, 6,499,463 pilgrims visited the Holy Masjid in 2021. Furthermore, according to Saudi Vision 2030, a significant increase in the number of pilgrims is expected in the coming years, with an estimated 30 million pilgrims visiting Umrah alone by 2030 [6,7].

Crowding and a variety of health risks, such as trauma and injury, can render MGs dangerous [1,3,5]. Additionally, the high concentration of people at specific locations for a set period increases the risk of spreading infectious diseases, including viral respiratory tract infections [3,4,8]. To control and prevent pulmonary system infections during MG events, regularly practicing proper hand hygiene (HH) is highly advised [3,4,8]. Good HH practice is considered one of the most effective strategies to halt the spread of infectious diseases. It has been shown that these precautions were crucial in managing the COVID-19 pandemic, potentially saving millions of lives [9,10].

While social distancing and avoiding close contact with others are challenging strategies to implement within the context of MGs, effective personal precautions can be taken to reduce the risk at a MG. These include using face masks, disposable handkerchiefs, cough etiquette, and maintaining proper HH [1,11]. Recognizing the critical role that hand cleanliness plays in preventing the transmission of infectious diseases, the WHO released guidelines in 2009 for HH practices in medical facilities [12,13]. However, studies focusing on infection prevention and control strategies at MGs are scarce. It is crucial to be aware of different hand-washing techniques and the appropriate times for washing hands in specific ways. High-quality education is required to achieve this goal [10,12].

The government of Saudi Arabia implemented a set of public health precautions during the recent Hajj and Umrah seasons to prevent the transmission of COVID-19 during the global pandemic [3,8]. These precautions included mandatory use of face masks, providing pilgrims with guidance on practicing frequent HH, supplying them with the proper sanitary supplies, and maintaining physical distance from others [3,8].

A previous study conducted in the city of Al Madinah Al Munawwarah intended to assess the HH awareness, perspectives, and behaviors of visitors to the Prophet’s Mosque reported a moderate score for HH knowledge, along with various knowledge gaps, particularly in relation to appropriate techniques for ensuring HH [1]. Another study conducted in Makkah Al-Mukarramah aimed at understanding the use of HH among Saudi pilgrims performing the Hajj demonstrated that HH was generally accepted among Saudi pilgrims. Nonetheless, there were knowledge gaps in several areas that could be addressed through health education and awareness campaigns [14].

No studies have specifically examined the level of HH awareness and the maintenance of proper HH among visitors to the Holy Masjid. Therefore, this research sought to evaluate the understanding among visitors to the Holy Masjid of adequate handwashing and proper HH habits during the month of Ramadan in 2023.

## Materials and methods

Study design and participants

A cross-sectional survey was conducted among visitors to the Holy Masjid (Masjid Al-Haram) in Makkah, Saudi Arabia, throughout the month of Ramadan in 2023, using a practical sampling technique. Both male and female visitors who were at least 18 years old were eligible to participate. However, minors, attendees younger than 18 years old, as well as those who were mentally incapable of comprehending the consent form in either English or Arabic were excluded.

Ethical considerations and sample size

After obtaining ethical approval from the Biomedical Ethics Committee of the College of Medicine at Umm Al-Qura University, Makkah, Saudi Arabia (Institutional Review Board reference number: HAPO-02-K-012-2023-04-1595), a self-administered electronic questionnaire was distributed in May 2023 via social media platforms to collect data. The OpenEpi website (version 3.01) was utilized to determine the sample size, providing a 95% confidence interval [15]. The minimum required sample size was calculated to be 385 participants. However, to ensure accuracy and enhance the generalizability of the results, 690 responses were collected.

Study tool and scoring

The questionnaire was developed using exemplary questions from a published survey [14]. The final manuscript was edited, and any grammatical mistakes in either Arabic or English were corrected. The questionnaire consisted of four sections: 1) the consent form, 2) demographic data of participants, 3) true/false statements to assess the research population’s understanding of common misconceptions, risks, and behaviors related to HH (each participant received a total knowledge score ranging from 0 to 12, where higher scores indicated adequate knowledge of HH, and lower scores suggested inadequate knowledge), and 4) self-reported items on HH behavior, such as the frequency of hand cleansing with various HH products (e.g., alcohol-based hand sanitizer, soap and water, and water only). Participants were asked about their use of these items before and after using the restroom, eating, and other activities likely to involve germ exposure.

Statistical analysis

After data collection, data were reviewed and fed into SPSS version 21 (IBM Corp., Armonk, NY, USA). All statistical methods were two-tailed, with an alpha level of 0.05; a P value of less than or equal to 0.05 was considered significant. The total level of a participant’s knowledge about HH was evaluated by adding up the discrete scores for different correct awareness items. The total score relative to knowledge about HH was classified as poor if the participant’s score was below 60% of the maximum score. A score was classified as a participant having a good level of knowledge if the participant’s score exceeded 60% of the overall score. Descriptive analysis was done by distribution and percentage of prescribing frequency for research variables including participant’s data, medical history, and employment. Also, participants’ knowledge and practices regarding HH were recorded, and general knowledge was graphed. Cross-tabulation was used to present factors associated with participants' awareness of HH and to test the relationship between their knowledge level and practices, with a chi-square test for significance and an exact probability test for small frequency distributions.

## Results

In total, 690 visitors to the Holy Masjid, who were eligible for the study, completed the questionnaire. The age of participants ranged from 18 to 65 years, with a mean age of 26.5 ± 13.9 years. Of these, 415 (60.1%) were female, and 580 (84.1%) were Saudi nationals. A total of 419 (60.7%) were single, while 248 (35.9%) were married. Additionally, 667 (96.7%) resided in Saudi Arabia. Regarding educational levels, 412 (59.7%) were university graduates, and 215 (31.2%) had completed secondary education. A total of 36 (5.2%) reported hypertension (HTN), 37 (5.4%) were diabetic, 21 (3%) had chronic respiratory diseases, and 597 (86.5%) reported no chronic health conditions. Of the 690 participants, 277 (40.1%) were students, 146 (21.2%) were unemployed, and 267 (38.7%) were employed. The monthly salary was reported as SR 5,000 by 127 participants (18.4%), SR 6,000-10,000 by 148 (21.4%), and SR 11,000-20,000 by 125 (18.1%). The remaining 134 (19.4%) reported no monthly income at all (Table 1).

**Table 1:** Socio-demographic data of visitors of Holy Masjid (Masjid Al-Haram) during Ramadan month in the year 2023.

Socio-demographic data	No.	%
Age in years		
18-24	295	42.8%
25-34	202	29.3%
35-44	103	14.9%
45-54	70	10.1%
55-65	20	2.9%
Gender		
Male	275	39.9%
Female	415	60.1%
Nationality		
Saudi	580	84.1%
Non-Saudi	110	15.9%
Marital status		
Single	419	60.7%
Married	248	35.9%
Divorced / widow	23	3.3%
Are you resident of Saudi Arabia?		
Yes	667	96.7%
No	23	3.3%
Educational level		
Below secondary	12	1.7%
Secondary / diploma	215	31.2%
University	412	59.7%
Post-graduate	51	7.4%
Do you have chronic disease		
None	597	86.5%
Hypertension	36	5.2%
Diabetes mellitus	37	5.4%
Chronic respiratory disease	21	3.0%
Chronic gastrointestinal disease	12	1.7%
Chronic heart disease	9	1.3%
Employment		
Unemployed / retired	146	21.2%
Student	277	40.1%
Employed	267	38.7%
Monthly income		
No monthly income	134	19.4%
5000 SR or less	127	18.4%
6000-10000 SR	148	21.4%
11000-15000 SR	125	18.1%
16000-20000 SR	88	12.8%
> 20000 SR	68	9.9%

In terms of their knowledge and practices of HH, 625 (90.6%) participants were aware that eye diseases could be transmitted due to poor HH. A total of 613 (88.8%) understood that skin infections could be transmitted through poor HH. Moreover, 593 (85.9%) reported that respiratory diseases, such as influenza, could be transmitted due to poor HH, and 567 (82.2%) were aware that hand, foot, and mouth disease could be transmitted by poor HH. Conversely, 611 (88.6%) participants knew that poor HH does not transmit diabetes, and 480 (69.6%) were aware that HIV/AIDS cannot be transmitted through poor HH. Additionally, 504 (73%) participants reported knowing that the body’s immunity is not diminished by consistent hand-washing (Table 2).

**Table 2:** Hand hygiene knowledge among visitors of Holy Masjid (Masjid Al-Haram) during Ramadan month, 2023.

Hand hygiene knowledge	True		False	
	No.	%	No.	%
Diarrheal disease can be transmitted due to poor hand hygiene	522	75.7%	168	24.3%
Respiratory diseases such as the flu can be transmitted due to poor hand hygiene	593	85.9%	97	14.1%
Hand, foot and mouth disease can be transmitted due to poor hand hygiene	567	82.2%	123	17.8%
HIV/AIDS can be transmitted due to poor hand hygiene	210	30.4%	480	69.6%
Skin infections can be transmitted due to poor hand hygiene	613	88.8%	77	11.2%
Eye infections can be transmitted due to poor hand hygiene	625	90.6%	65	9.4%
Diabetes can be transmitted due to poor hand hygiene	79	11.4%	611	88.6%
Maintaining constant hand hygiene reduces the body's immunity	186	27.0%	504	73.0%
Alcohol-based hand sanitizer that contains 40% alcohol is sufficient to disinfect hands from germs	282	40.9%	408	59.1%
Rubbing hands until soap forms a lather for 10s before rinsing is enough for hand disinfection	429	62.2%	261	37.8%
Hands should be held under water while lathering with soap	227	32.9%	463	67.1%
The water temperature does not affect the hygiene result of hand washing	360	52.2%	330	47.8%

**Figure 1:**
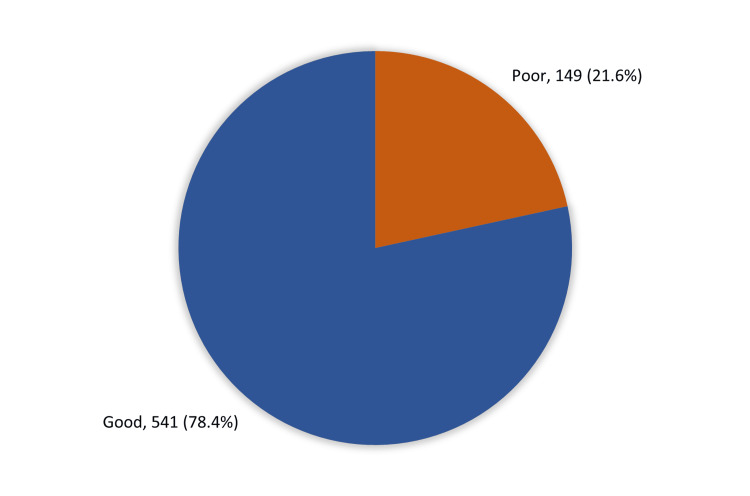
Overall hand hygiene knowledge among visitors of Holy Masjid (Masjid Al-Haram) during Ramadan month, 2023.

Figure 1 shows the overall HH knowledge of the visitors to the Holy Masjid during the month of Ramadan in 2023. A total of 541 (78.4%) participants had good overall knowledge regarding HH, while 149 (21.6%) had poor knowledge.

Table 3 reveals that 512 (74.2%) participants used soap and water to clean their hands before meals, while 9 (1.3%) used an alcohol-based hand sanitizer. After eating, 599 (86.8%) used soap and water for hand cleaning. Similarly, 596 (86.4%) used soap and water after using the restroom. Regarding other activities, 299 (43.3%) cleaned their hands with soap and water after caring for a sick person, 235 (34.1%) after sneezing or coughing, and 639 (92.6%) when their hands were visibly dirty. Finally, 539 (78.1%) reported using soap and water to clean their hands after disposing of a garbage bag, whereas 282 (40.9%) used nothing to clean their hands after shaking hands with someone.

**Table 3:** Hand hygiene practice among visitors of Holy Masjid (Masjid Al-Haram) during Ramadan month, 2023.

Practice	Water only		Water and soap		Alcohol-based hand rub		Wet wipes		None	
	No.	%	No.	%	No.	%	No.	%	No.	%
The most common method you use to clean hands before meal	132	19.1%	512	74.2%	9	1.3%	8	1.2%	29	4.2%
The most common method you use to clean hands after meal	61	8.8%	599	86.8%	9	1.3%	16	2.3%	5	.7%
The most common method you use to clean hands after toilet use	58	8.4%	596	86.4%	22	3.2%	6	.9%	8	1.2%
The most common method you use to clean hands after caring for sick person	23	3.3%	299	43.3%	328	47.5%	7	1.0%	33	4.8%
The most common method you use to clean hands after sneezing/ coughing	109	15.8%	235	34.1%	104	15.1%	107	15.5%	135	19.6%
The most common method you use to clean hands when your hands are visibly dirty	15	2.2%	639	92.6%	20	2.9%	11	1.6%	5	.7%
The most common method you use after disposal of garbage bag	51	7.4%	539	78.1%	45	6.5%	27	3.9%	28	4.1%
The most common method you use after shaking hands	51	7.4%	144	20.9%	178	25.8%	35	5.1%	282	40.9%

In terms of the factors associated with participants’ knowledge about HH, 338 (81.4%) female participants had overall good knowledge about HH, versus 203 (73.8%) males. The recorded statistical significance was P = 0.017. Also, 125 (85.6%) unemployed participants had an overall good level of knowledge about HH compared to 202 (75.7%) employed participants (P = 0.048). All other factors showed an insignificant association with participants’ level of knowledge regarding HH (Table 4).

**Table 4:** Factors associated with participants' knowledge about hand hygiene. $: Exact probability test * P < 0.05 (significant)

Socio-demographic data	Knowledge level				P-value
	Poor		Good		
	No.	%	No.	%	
Age in years					0.105^$^
18-24	64	21.7%	231	78.3%	
25-34	46	22.8%	156	77.2%	
35-44	28	27.2%	75	72.8%	
45-54	7	10.0%	63	90.0%	
55-65	4	20.0%	16	80.0%	
Gender					0.017*
Male	72	26.2%	203	73.8%	
Female	77	18.6%	338	81.4%	
Nationality					0.230
Saudi	130	22.4%	450	77.6%	
Non-Saudi	19	17.3%	91	82.7%	
Marital status					.373
Single	94	22.4%	325	77.6%	
Married	48	19.4%	200	80.6%	
Divorced / widow	7	30.4%	16	69.6%	
Are you resident of Saudi Arabia?					0.618^$^
Yes	145	21.7%	522	78.3%	
No	4	17.4%	19	82.6%	
Educational level					0.188
Below secondary	1	8.3%	11	91.7%	
Secondary / diploma	51	23.7%	164	76.3%	
University	91	22.1%	321	77.9%	
Post-graduate	6	11.8%	45	88.2%	
Employment					0.048*
Unemployed / retired	21	14.4%	125	85.6%	
Student	63	22.7%	214	77.3%	
Employed	65	24.3%	202	75.7%	
Monthly income					0.122
No monthly income	26	19.4%	108	80.6%	
5000 SR or less	34	26.8%	93	73.2%	
6000-10000 SR	24	16.2%	124	83.8%	
11000-15000 SR	29	23.2%	96	76.8%	
16000-20000 SR	25	28.4%	63	71.6%	
> 20000 SR	11	16.2%	57	83.8%	
Do you have chronic disease?					0.624^$^
None	124	20.8%	473	79.2%	
Hypertension	9	25.0%	27	75.0%	
Diabetes mellitus	8	21.6%	29	78.4%	
Chronic respiratory disease	6	28.6%	15	71.4%	
Chronic gastrointestinal disease	4	33.3%	8	66.7%	
Chronic heart disease	3	33.3%	6	66.7%	

Analyzing the relationship between participants’ knowledge about HH and their actual hygiene practices, 530 (76.8%) participants with good knowledge about HH cleaned their hands before meals, compared to 131 (19%) with poor knowledge (P = 0.001). Moreover, 539 (78.1%) of those with good knowledge cleaned their hands after meals, versus 146 (21.6%) with poor knowledge (P = 0.754). In addition, 540 (78.3%) participants with good HH knowledge, versus 142 (20.6%) with poor knowledge, cleaned their hands after using the restroom (P = 0.001). Furthermore, 521 (75.5%) with good HH knowledge cleaned their hands after caring for a sick person, compared to 136 (19.7%) with poor knowledge (P = 0.011). Lastly, 540 (78.3%) with good HH knowledge cleaned their hands when they were visibly dirty, versus 145 (21%) with poor knowledge (P = 0.001) (Table 5).

**Table 5:** Relation between participants' knowledge about hand hygiene and their practice. *P < 0.05 (significant).

Hand hygiene practice	Knowledge level				P-value
	Poor		Good		
	No.	%	No.	%	
Clean hands before meal					0.001*
Yes	131	87.9%	530	98.0%	
No	18	12.1%	11	2.0%	
Clean hands after meal					.745
Yes	146	98.0%	539	99.6%	
No	3	2.0%	2	.4%	
Clean hands after toilet use					.001*
Yes	142	95.3%	540	99.8%	
No	7	4.7%	1	.2%	
Clean hands after caring for sick person					0.011*
Yes	136	91.3%	521	96.3%	
No	13	8.7%	20	3.7%	
Clean hands after sneezing/coughing					0.462
Yes	123	82.6%	432	79.9%	
No	26	17.4%	109	20.1%	
Clean hands when your hands are visibly dirty					0.001*
Yes	145	97.3%	540	99.8%	
No	4	2.7%	1	0.2%	
Clean hands after disposal of garbage bag					0.360
Yes	141	94.6%	521	96.3%	
No	8	5.4%	20	3.7%	
Clean hands after shaking hands					0.440
Yes	84	56.4%	324	59.9%	
No	65	43.6%	217	40.1%	

## Discussion

HH is a crucial factor in preventing the spread of diseases and infections, particularly in MGs. To our knowledge, our study is the first to assess the understanding of HH and proper HH habits among visitors to the Holy Masjid in 2023 during the month of Ramadan. In line with our expectations based on related literature, our data revealed that a majority of the participants, 541 (78.4%), were knowledgeable about HH. However, other studies have indicated that their participants had a mediocre level of knowledge about HH, possibly due to their smaller sample sizes compared to our larger sample [1,5,14]. Most respondents in our study were fully aware of the benefits that HH can offer in preventing common diseases, such as gastrointestinal and respiratory system infections. These results support the findings of the Mahdi H et al. study [14].

Another study reported that 40% of participants knew very little about how HH could prevent hand, foot, and mouth disease [14]. Conversely, in our study, most participants demonstrated good knowledge of this connection. It was not surprising to find that some pilgrims in our study lacked education or knowledge regarding more specialized topics, such as the necessity for hand sanitizers to contain alcohol and the appropriate duration for applying hand sanitizer. However, pilgrims to the Holy Masjid appeared to possess more knowledge than community members in other industrialized countries in Asia [16].

Most of the respondents indicated that they did not believe that keeping the hands clean would reduce the body’s immunity, which contradicted a similar study that indicated most respondents believed that consistently keeping the hands clean would weaken the body’s natural defenses [17]. Our findings on the prevalence of HH with soap and water align with research on HH practices among pilgrims in Saudi Arabia. Most people washed their hands with soap and water, consistent with prior research [5,14]. Our results demonstrate comparable HH practices. Notably, 299 (43.3%) of participants engaged in HH after caring for an ill individual, and 235 (34.1%) after coughing or sneezing. These findings align with those of a separate study on pilgrims in Saudi Arabia, which reported HH rates of 39.7% after contact with a sick person and 31.9% after coughing or sneezing [14]. In both studies, the results showed relatively low HH behavior related to illness, whether it was the participant who was sick or when the participant had encountered a sick person.

Study limitations 

One limitation of our study was the availability of the questionnaire only in Arabic and English. This could have introduced bias due to language barriers. During the Umrah season in Ramadan, many pilgrims come from diverse nationalities with varied languages. Therefore, excluding languages other than Arabic and English might have excluded responses from a significant portion of the pilgrim population.

## Conclusions

The purpose of this study was to evaluate the understanding of HH and proper HH habits among visitors to the Holy Masjid during Ramadan in 2023. The study discovered that a significant majority of participants, 541 (78.4%), demonstrated a robust knowledge of HH, particularly its effectiveness in preventing respiratory and systemic infections. However, deficiencies were noted in their understanding of alcohol-based hand sanitizers and the optimal duration for their application. A notable portion of participants also lacked awareness of the relationship between HH and immune modulation. Behaviorally, post-caretaking HH was observed in only 299 (43.3%) of participants, and after sneezing or coughing, this practice was reported by just 235 (34.1%), indicating an area for improvement in HH practices.

Participants with good knowledge were significantly more likely to clean their hands before eating, after using the restroom, after caring for a sick person, and when their hands were visibly dirty. The study's findings highlight the need for improved HH knowledge and practices among visitors to the Holy Masjid, advocating for sustained and intensive awareness-raising interventions.
